# Effect of Adventitial Dissection of Superficial Temporal Artery on the Outcome of Superficial Temporal Artery-Middle Cerebral Artery Bypass in Moyamoya Disease

**DOI:** 10.14336/AD.2016.1115

**Published:** 2017-07-21

**Authors:** Xin Li, Zheng Huang, Ming-Xing Wu, Dong Zhang

**Affiliations:** ^1^Department of Neurosurgery, Beijing Tiantan Hospital, Capital Medical University, Beijing 100050, China; ^2^Department of Neurosurgery, Xiangya Hospital, Central South University, Changsha 410008, Hunan, China; ^3^Department of Neurosurgery, Beijing Puhua International Hospital, Beijing 100050, China.

**Keywords:** Moyamoya disease, STA-MCA bypass, anastomosis, Laser Doppler Flowmetry, Hemodynamics, cerebral revascularization, adventitia, ischemia

## Abstract

Superficial temporal artery-middle cerebral artery (STA-MCA) has been used for the treatment of occlusive cerebrovascular disease including moyamoya disease. The effect of STA-MCA bypass depends not only on the patency of anastomosis, but also on integrity and functional capacity of the donor artery. In the present prospective study, we investigated the effect of extensive stripping STA adventitia and fasciae on hemodynamic function in STA-MCA bypass of moyamoya disease patients. Twenty patients (n=8 in control group, n=12 in stripping group) of moyamoya disease were subjected to STA-MCA end-to-side direct anastomosis. Perfusion unit (PU) values of the cortex were measured and recorded using a Laser Doppler flowmetry (LDF) for 5 days. Computed tomography perfusion was performed to determine blood flow before and after bypass. No patient experienced significant neurologic deficits associated with neurosurgical complications. LDF demonstrated that adventitial stripping group had higher cerebral blood flow increase than control group. The adventitia stripping group tends to have higher rate of increased cerebral perfusion after bypass than non-stripping group. Furthermore, the ultrasound examination at 3 days after bypass demonstrated that the adventitial stripping group has a tendency of bigger STA and higher peak systolic velocity than control group. Our result suggests that stripping adventitia of STA improves hemodynamics of STA-MCA bypass in moyamoya disease.

Superficial temporal artery-middle cerebral artery (STA-MCA) bypass surgery is a technique that allows low blood flow supply from the extracranial carotid to the distal MCA [1, 2]. STA-MCA bypass procedure has been adopted for the treatment of cerebrovascular occlusive disease and as an adjunct treatment for complex intracranial aneurysms [1]. The key of STA-MCA bypass depends on not only the anastomosis patency but also the blood flow of the donor artery. STA travels within the temporal muscle fascia, thus, its expansion could be affected mechanically by the adventitia and surrounding fascia. In addition, adventitia and fascia of STA contained a large amount of sympathetic nerve fibers [3]. The sympathetic nerve of the artery may play an important role in vascular tone thus affect the blood flow after the STA-MCA bypass. We expected that removal of the sympathetic nerve fibers of the STA may promote the artery dilation hence increase blood flow after anastomosis. The present study was to investigate whether stripping of STA adventitia and surrounding fascia has an effect on the bypass patency and regional cerebral blood flow.

**Table 1:** Patient’s information.

Age (years)	Sex	Preop presentation	Surgery side	Stripping	ICGA	DSA	CTA	Postop rCBF	Postop result	Follow-up mRS		Follow-up cerebrovascular events
										Postop	Current	
25	F	SAH	L	Yes	++	++	++	Improved	Excellent	0	0	No
40	M	Ventricular hemorrhage	L	Yes	+		+	Improved	Excellent	0	0	No
41	M	Infarction, TIA	R	Yes		+	+	Unchanged	Good	1	1	Mild headache
45	F	Infarction, TIA	L	Yes		+	+	Improved	Excellent	2	2	No
46	F	Infarction, TIA	L	Yes		+	+	Improved	Good	0	0	No
25	M	Infarction, TIA	R	Yes	+	+	+	Improved	Good	0	0	No
51	M	TIA	R	Yes		+		Improved	Excellent	0	0	No
48	F	Infarction, TIA	R	Yes		+	+	Improved	Good	0	0	Mild headache
52	F	Infarction, TIA	R	Yes	+	++	++	Improved	Good	0	0	No
42	F	TIA	L	Yes			+	Unchanged	Good	0	1	TIA 3 years postop
41	F	Infarction, TIA	L	Yes	+		+	Unchanged	Excellent	0	0	No
57	M	TIA	L	Yes	++	++	++	Improved	Excellent, CHS	0	0	No
43	M	Infarction, TIA	R	No	++	++	++	Improved	Excellent	1	1	No
29	F	TIA	L	No	+	++		Unchanged	Good	0	0	No
22	M	Infarction, TIA	L	No	++	+		Improved	Good	0	0	No
41	F	Infarction, TIA	L	No	+		+	Unchanged	Excellent	1	1	No
34	M	TIA	L	No	++		++	Improved	Excellent	0	0	No
40	F	Infarction, TIA	R	No	+	+	++	Improved	Good	0	1	TIA 3 years postop
47	M	Infarction, TIA	R	No	++	++	++	Improved	Good, CHS	0	0	No
38	F	Frontal hemorrhage into ventricles	R	No			+	Unchanged	Excellent	0	1	Mild headache

*CHS*, cerebral hyperperfusion syndrome; *CTA*, computed tomographic arteriography; *DSA*, digital subtraction angiography; *F*, female; *ICGA*, indocyanine green angiography; *L*, left; *M*, male; *mRS*, modified Rankin Scale; *Postop*, postoperative; *Preop*, preoperative; *R*, right; *rCBF* regional cerebral blood flow; *SAH*, subarachnoid hemorrhage; *STA-MCA*, superficial temporal artery-middle cerebral artery; *TIA*, transient ischemic attack; *+*, patent; *++*, highly patent.

## MATERILAS AND METHODS

### Patients and clinical features

Twenty patients of moyamoya disease underwent STA-MCA bypass at Beijing Tiantan Hospital from 2012 to 2013 were included in the present study, in which 9 were male and 11 were female (age range, 22 to 58 years; mean age, 41.19 years) (Table 1). The main symptoms were ischemic stroke in 17 and hemorrhagic stroke in 3 patients. All patients were diagnosed of moyamoya disease by digital subtraction angiography (DSA), magnetic resonance imaging (MRI), computed tomography (CT), and computed tomography perfusion (CTP).

### Surgical procedures

All patients were subjected unilateral or bilateral STA-MCA end-side anastomosis under general anesthesia. Before the end-side anastomosis of STA-MCA, the conjunctive and adventitial tissue of STA was dissected. For the adventitial dissection group (n=12), the length of dissection was 4-5 cm from the distal end of STA. Part of the donor vessel under the bone flap was unfolded to fit the bone flap and the dura to reduce the bending. There were 8 patients in the control group in which adventitial and conjunctive tissue were dissected and removed no more than 1 cm from distal end of STA [2]. The patency of anastomosis was determined using intraoperative near-infrared indocyanine green angiography (ICGA).

### Perioperative management

All patients underwent computed tomography angiography (CTA), CTP and/or DSA before and after bypass. The relative change of the perfusion parameter values was evaluated to determine whether CBF was increased after procedure. All patients were subjected to GE-LOGIQ9 color ultrasound examination using 7L transducer (bandwidth 2.5-7.0 MHz) (GE Healthcare, London, UK) to measure the hemodynamic parameters of the feeding artery before surgery and at 3 days after bypass, including peak systolic velocity (PSV), end-diastolic velocity (EDV), inner diameter (ID), and resistance index (RI).

rCBF was monitored using a LDF (Periflux 5010; Perimed, Beijing, China). Before anastomosis, a LDF probe was placed at different area of the cortex and rCBF was measured to identify the most severe ischemic area. After anastomosis, the LDF probe was placed to the same site of the cortex and implanted subcutaneously as described previously [4, 5]. The rCBF was measured before and immediately after STA-MCA bypass, and at 1, 2, 3, 4, and 5 days after surgery. The LDF probe was pulled out carefully at day 5 after surgery. The rCBF was defined as perfusion unit (PU) value.

The functional outcome assessment was performed using the modified Rankin Scale (mRS) at discharge. The patients were follow-up for transient ischemic attack (TIA), new infarction, and cerebral hemorrhage.

### Statistical analysis

Data are expressed as mean ± standard deviation and analyzed using independent sample t test with SPSS version 19.0 (SPSS, Chicago, IL). The improvement of PU value from the LDF measurement on the same time point between the two groups was analyzed by two-way ANOVA. The differences in ID, PSV, EDV, and RI from color ultrasound examination before and after the bypass were analyzed by t test. The difference in rCBF from CTP before and after the bypass was compared qualitatively using Chi-square tests. A *p* < 0.05 was considered statistically significant.

## RESULTS

### Follow-up

All twenty patients included in the current study had symptoms improvement within the first 2 weeks after surgery. Two patients had transient postoperative headache due to cerebral hyperperfusion syndrome. No other significant neurologic deficit was observed after surgery. All 20 patients were followed up from 59-92 months with a median duration of 66.6 months. No intracranial hemorrhage and infarction were observed during the follow-up. Two patients had TIA at 3 years after surgery. Mild headache occurred in 3 patients. During the follow-up period, mRS maintained at 0 in 12 patients, at 1 to 2 in 4 patients, and improved from 0 to 1 in 2 patients (Table 1).

**Table 2:** Summary of difference in PU value between the adventitial dissection and control groups.

Group	PU_postop_- PU_preop_	PU_1d-postop_- PU_preop_	PU_2d-postop_- PU_preop_	PU_3d-postop_- PU_preop_	PU_4d-postop_- PU_preop_	PU_5d-postop_- PU_preop_
Nonstripping (8)	44.70 ± 14.92	308.98 ± 92.20	336.45 ± 105.79	351.02 ± 114.83	338.62 ± 71.73	249.89 ± 82.04
Stripping (12)	42.70 ± 17.47	479.72 ± 63.49	447.90 ± 78.11	543.43 ± 80.07	457.51 ± 87.39	536.11 ± 92.57

Values are mean ± standard deviation. *PU_preop_*, mean of LDF value measured before bypass; *PU_postop_*, mean of LDF value measured immediately after bypass; *PU_1d-postop_*, mean of LDF value measured at 1 day after bypass; *PU_2d-postop_*, mean of LDF value measured at 2 days after bypass; *PU_3d-postop_*, mean of LDF value measured at 3 days after bypass; *PU_4d-postop_*, mean of LDF value measured at 4 days after bypass_;_ *PU_5d-postop_*, mean of LDF value measured at 5 days after bypass.

**Figure 1.**
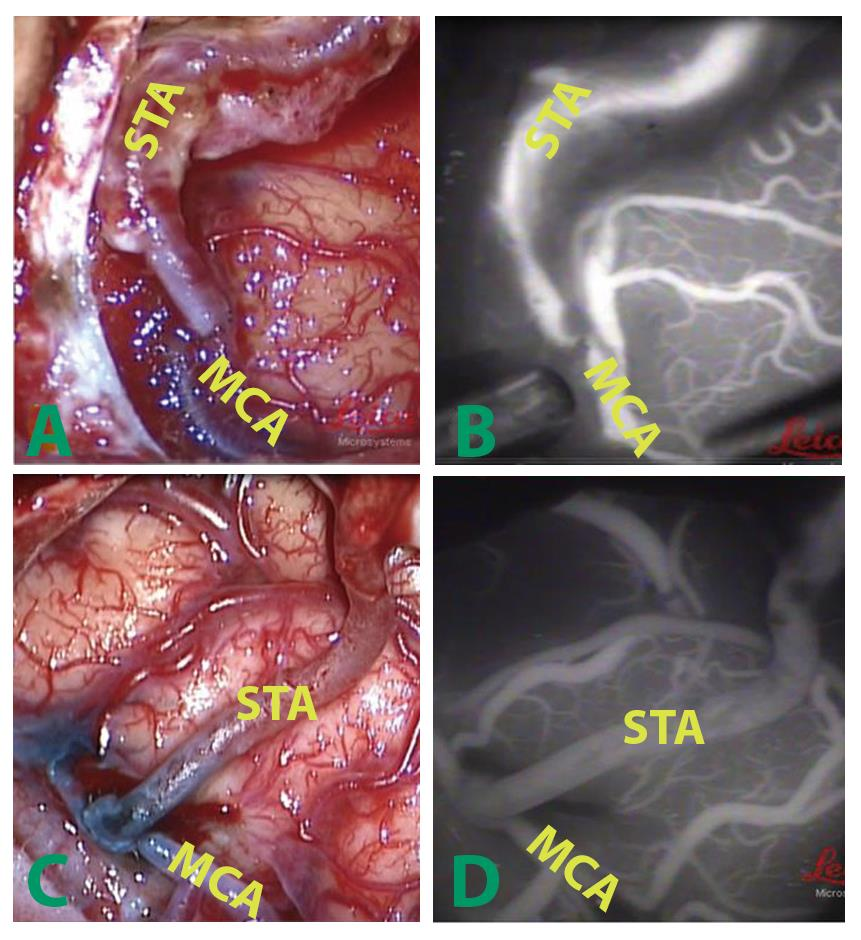
Intraoperative photo and ICGA image in adventitial stripping and control patients Representative intraoperative photo (**A, C**) and ICGA image (**B, D**) of STA-MCA bypass in non-adventitial stripping (**A, B**) and stripping (**C, D**) group. Extensive adventitial and facial stripping was shown in the adventitial stripping group (**C**) as compared with non-stripping group (**A**).

### Adventitial stripping improved blood perfusion after STA-MCA bypass as compared with non-dissection

All the 20 patients were subjected ultrasound, DSA and/or CTA to determine patency of the donor artery. Fourteen patients had intraoperative near-infrared ICGA (Fig. 1). CTP were performed in all 20 patients and increase of cerebral perfusion was observed in 62.5% (5/8) and 75% (9/12) of the control and adventitial stripping group, respectively.

**Figure 2.**
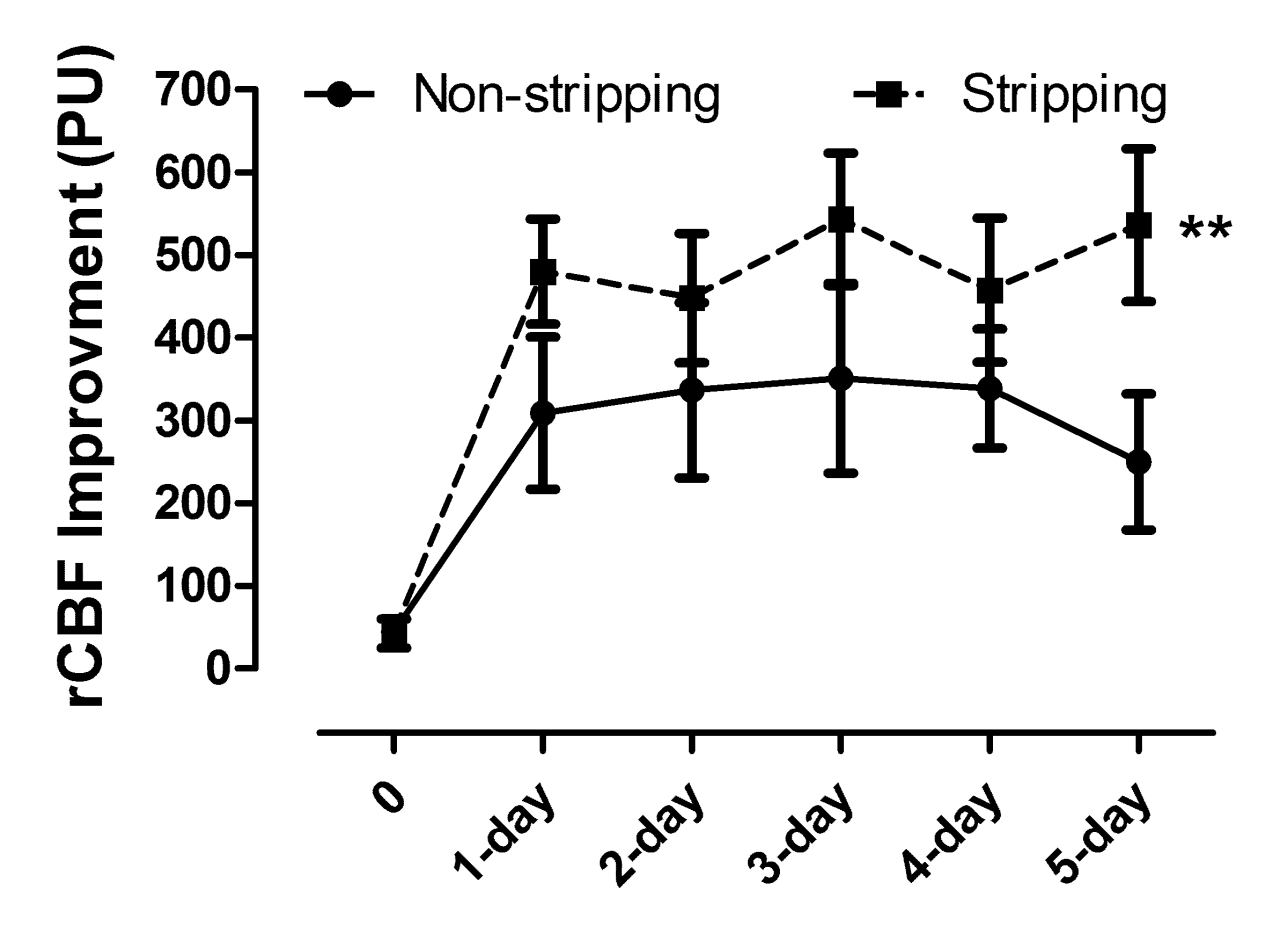
Improvement of rCBF after STA-MCA bypass in non-stripping and stripping group Significant improvement of rCBF was indicated in both non-stripping and stripping groups. In addition, stripping adventitia and facial tissue significant improved the rCBF (2-way ANOVA, *p*<0.01).

The mean baseline rCBF determined by LDF in the area of anastomosis was 49.30 ± 19.22 and 62.61 ± 27.78 in the adventitial dissection and control group, respectively (p = 0.220). PU values were significantly elevated in both the adventitial stripping and control groups, especially at first day after surgery. Stripping adventitia and facial tissue significant improved the rCBF (2-way ANOVA, p<0.01) (Table 2 and Fig. 2).

We analyzed the change of hemodynamic parameters obtained by ultrasound between the two groups. The improvement of ID, PSV, EDV, and RI was higher in the adventitial stripping group than non-dissection group, although no statistical difference was observed (Table 3).

## DISCUSSION

STA-MCA bypass allows blood flow bypass proximal lesion of intracranial vasculature and has been used for occlusive cerebrovascular disease for almost half century [6]. In 1980s, STA-MCA bypass fell from favor after an international randomized trial failed to demonstrate any benefit of extracranial-intracranial arterial bypass to reduce the risk of ischemic stroke [7]. In the last two decades, the modern technological advancements such as LDF [4, 5, 8], infrared camera thermography [8], and quantitative MRA [9] for hemodynamic measurement have allowed us to reappraise the effect of STA-MCA bypasses on cerebrovascular disease [10, 11]. There is increasing evidence indicated that STA-MCA bypasses not only improve neurological function but also prevent stroke recurrence in subgroup patients of cerebrovascular disease [12-18].

Moyamoya disease is a cerebrovascular disorder characterized by chronic progressive stenosis of intracranial internal carotid arteries and their proximal branches that predisposes the affected patients to stroke. While originally considered exclusive to East Asia, particular in Japan, moyamoya disease has been increasingly diagnosed all over the world with two peaks of age distribution at 5 years and about 40 years [19]. The direct revascularization via STA-MCA anastomosis provides immediate blood supply for the treatment of moyamoya disease. Large-scale case studies have indicated that STA-MCA anastomosis usually has very high bypass patency rate (>90%) [20, 21]. Nonetheless, the improvement of blood flow after STA-MCA bypass has been found inconsistent due to various factors, including age of patients, the length of donor vessel, temporal muscle compression, and graft spasm [22-24] [4] [25] [26, 27].

The success of the revascularization of STA-MCA bypass is greatly depended on the integrity and functional capacity of the donor artery, STA. The cerebrovascular bed is well innervated by both sympathetic and parasympathetic nerve fibers. Although the role of the autonomic nerve system in the regulation of cerebral blood flow remains controversial, it has been indicated that decreasing sympathetic tone may offer therapeutic benefit during cerebral vasospasm [28]. A large amount of sympathetic nerve fibers and nerve endings have been found in STA adventitia and fascia [3, 29]. We speculated that the sympathetic control of STA contributes to the hemodynamic instability after STA-MCA bypass. The present study was to determine the effect of STA denervation on the outcome of STA-MCA bypass in moyamoya disease patients.

**Table 3:** Summary of color Doppler hemodynamic parameters between the adventitial dissection and control groups at 3 days after bypass.

Group	ID_postop_- ID_preop_	PSV_postop_- PSV_preop_	EDV_postop_- EDV_preop_	RI_preop_- RI_postop_
Nonstripping (8)	0.338 ± 0.068	28.63 ± 6.38	20.76 ± 3.86	0.1425 ± 0.1666
Stripping (12)	0.408 ± 0.083	44.17 ± 8.39	25.75 ± 5.76	0.1600 ± 0.0329
*P*	0.550	0.196	0.528	0.641

Values are mean ± standard deviation. *EDV*, mean of end-diastolic velocity; *ID*, inner diameter; *PSV*, peak systolic velocity; *RI*, resistance index; *postop*, value measured after bypass surgery; *preop*, value measured before bypass surgery.

For STA-MCA bypass, periadventitial tissue layers of STA are normally kept intact with removing conjunctive and adventitial tissues only within 1 cm of the distal end for anastomosis [30] [2, 31, 32]. In the present study, denervation was achieved by removal adventitia and surrounding fascia at 4-5 cm of the distal end of STA. Our results demonstrated that stripping adventitial tissues may improve hemodynamic function of STA, evidenced by the increased rCBF measured by LDF. Consistently, the adventitia stripping group tends to have higher rate of increased cerebral perfusion after bypass than non-stripping group. Furthermore, the ultrasound examination at 3 days after bypass demonstrated that the adventitial stripping group has a tendency of bigger STA and higher peak systolic velocity than control group.

Cerebral hyperperfusion syndrome has been well recognized after STA-MCA in moyamoya patients, characterized by ipsilateral throbbing headache, ocular and facial pain, seizure, and focal neurological deficits [33, 34]. In addition, cerebral hyperperfusion has been found to be associated with intracranial hemorrhage after STA-MCA bypass [35]. Direct STA-MCA surgery is a low-flow bypass with less possibility of hyperperfusion [27]. Indeed, hypoperfusion, but not hyperperfusion, has been found to be associated with the transient neurological events in moyamoya disease patients after STA-MCA bypass [36]. In the present study, cerebral hyperperfusion syndrome was only observed in 2 patients presented as transient postoperative headache.

In summary, our study has provided the first evidence that stripping the donor artery of adventitia and fascia might improve the hemodynamic function of STA-MCA bypass. We expect that the extensive stripping of adventitia and fascia in the donor artery denervates sympathetic control of STA, further inhibits the potential vasospasm after bypass procedure. Our promising result warrants further larger scale clinical study to determine the effect of extensive adventitia and fascia stripping on the long-term outcome of STA-MCA bypass in moyamoya disease patients.
